# The Geminin and Idas Coiled Coils Preferentially Form a Heterodimer That Inhibits Geminin Function in DNA Replication Licensing*

**DOI:** 10.1074/jbc.M113.491928

**Published:** 2013-09-24

**Authors:** Christophe Caillat, Dafni-Eleftheria Pefani, Peter J. Gillespie, Stavros Taraviras, J. Julian Blow, Zoi Lygerou, Anastassis Perrakis

**Affiliations:** From the ‡ Division of Biochemistry, The Netherlands Cancer Institute, 1066 CX Amsterdam, The Netherlands,; § Laboratory of Biology, School of Medicine, University of Patras, 26505 Rio, Patras, Greece,; ¶ Centre for Gene Regulation and Expression, College of Life Sciences, University of Dundee, Dundee DD1 5EH, United Kingdom, and; ‖ Laboratory of Physiology, School of Medicine, University of Patras, 26505 Rio, Patras, Greece

**Keywords:** DNA Replication, Isothermal Titration Calorimetry, Protein Stability, X-ray Crystallography, X-ray Scattering, Xenopus, Coiled Coil

## Abstract

Geminin is an important regulator of proliferation and differentiation in metazoans, which predominantly inhibits the DNA replication licensing factor Cdt1, preventing genome over-replication. We show that Geminin preferentially forms stable coiled-coil heterodimers with its homologue, Idas. In contrast to Idas-Geminin heterodimers, Idas homodimers are thermodynamically unstable and are unlikely to exist as a stable macromolecule under physiological conditions. The crystal structure of the homology regions of Idas in complex with Geminin showed a tight head-to-head heterodimeric coiled-coil. This Idas-Geminin heterodimer binds Cdt1 less strongly than Geminin-Geminin, still with high affinity (∼30 nm), but with notably different thermodynamic properties. Consistently, in *Xenopus* egg extracts, Idas-Geminin is less active in licensing inhibition compared with a Geminin-Geminin homodimer. In human cultured cells, ectopic expression of Idas leads to limited over-replication, which is counteracted by Geminin co-expression. The properties of the Idas-Geminin complex suggest it as the functional form of Idas and provide a possible mechanism to modulate Geminin activity.

## Teaser

**Background:** Idas is a homologue of the proliferation and differentiation regulator, Geminin.

**Results:** Idas and Geminin form preferentially a stable heterodimeric coiled-coil that interacts with Cdt1 *in vitro* and in cells.

**Conclusion:** The Idas-Geminin complex inhibits Geminin function in DNA replication licensing.

**Significance:** The Idas-Geminin complex, possibly the only functional form of Idas, likely regulates the diverse functions of Geminin.

## Introduction

Several overlapping pathways control timely DNA replication (1–3). The relative importance of these regulatory mechanisms differs, depending on the cell type and stage of development. An important step for DNA replication is the assembly of pre-replicative licensing complexes on origins of replication. These multiprotein complexes contain the origin recognition complex and the loading factors Cdc6 and Cdt1, which direct loading of the six subunit replicative helicase (MCM2–7) onto origin DNA, licensing chromatin for replication. The regulation of pre-replicative complex formation is crucial for once per cell cycle replication (4, 5). It involves control of the expression level, inactivation by cyclin-dependent protein kinases (CDK), and cell cycle-specific degradation of pre-replicative complex components. Although these mechanisms are conserved across eukaryotes, fine-regulation may vary (6). In metazoans, Cdt1 constitutes a major determinant of timely DNA replication licensing, and a divergence point introduced a new mechanism for Cdt1 regulation by the Geminin protein (7–9). This additional mechanism appears essential to coordinate cell proliferation to differentiation processes in metazoans (10, 11).

Geminin is a small protein that can be structurally divided in three domains (12). The N-terminal domain (1–95 in human Geminin) is most likely intrinsically disordered and contains a destruction box (residues 23–31) recognized by the APC/C and likely also a neuralizing domain (residues 38–90) identified as sufficient to induce uncommitted embryonic cells to differentiate into neurons in early *Xenopus* development (13). Similarly, the C-terminal domain (160–209) appears to be also unstructured and mediates the interaction with the Brahma (Brm) catalytic subunit of the SWI/SNF chromatin remodeling complex (14, 15). The same domain (171–190) is responsible for the interaction with homeodomain-containing regulators of DNA transcription (16) such as Six3 (17) and Hox family members (18), previously proposed to take place through the coiled coil region. The central, structured, domain (96–159) assembles in a head-to-head coiled coil homodimer in solution (19), and the Geminin homodimer binds Cdt1 with a high affinity (8, 20). The structure of Geminin in complex with Cdt1 showed that in the Geminin coiled coil dimer, N-terminal residues bind tightly to Cdt1 (20, 21). A third, more labile, interface mediating Cdt1 binding is in the C-terminal region of the dimeric Geminin coiled coil (21) and leads to dimerization of the Geminin-Cdt1 complex, to form a heterohexamer. A role of different stoichiometric complexes has been proposed for the mechanism enabling Geminin to inhibit binding of Cdt1 to the MCM complex and recruit it to the origin recognition complex-Cdc6-chromatin sites (21, 22). Along all these interactions, the coiled-coil domain of Geminin has also been suggested to interact with other coiled-coil-containing proteins implicated in cell fate decisions, such as ERNI and BERT, which are implicated in neural plate acquisition (23) and Idas (24).

Idas was recently identified as a Geminin-related protein, which interacts with Geminin and exhibits high levels of expression in the choroid plexus and the cortical hem of the developing mouse forebrain (24). Idas regulates different aspects of the cell cycle, as its depletion causes abnormal S phase progression, and its overexpression results in multipolar spindle formation (24). Idas (called Multicilin in a subsequent study; Stubbs *et al.* (25)) has also been identified as a key regulator of multiciliate cell differentiation in diverse tissues by coordinately promoting cell cycle exit, centriole assembly, and FoxJ1 expression (25). Like Geminin, Idas may, therefore, participate in both cell cycle control and cell fate decisions. Idas interacts with Geminin through the coiled-coil domain. We have previously demonstrated that Idas prefers to interact with Geminin than with itself (24), but the mechanism defining this interaction remained unclear.

In this study we focus on the mechanism of Geminin and Idas interaction and its implication for the function of both proteins in DNA replication. We determined the crystal structure of the complex between the coiled-coil domain of Geminin and Idas (tGeminin and tIdas) and analyzed the stability of possible Geminin and Idas complexes. Functional data in *Xenopus* egg extracts and mammalian cells combined with a thermodynamic analysis of the association of these complexes with Cdt1 imply that Idas functions in cells as a heterodimer with Geminin and can modulate its activity.

## EXPERIMENTAL PROCEDURES

### 

#### 

##### Cloning, Expression, and Purification

The constructs dIdas (101–284), dGeminin (29–209), and the coiled-coil domain tIdas (173–245) and tGeminin (82–160) were cloned into the pETNKI-His-3CLIC-kan vector (26) for expression with a cleavable His tag or into the pET22b (Novagen) vector for expression without tag. As these two plasmids are resistant to kanamycin and ampicillin, respectively, they allow efficient co-expression experiments. Cdt1 (115–353) was cloned into the pCOLDNKI-His-TF-3C-LIC-amp vector (26) to prevent degradation in absence of Geminin. The tIdas-tGeminin complex was finally purified by size exclusion chromatography in a buffer containing 50 mm HEPES/NaOH pH 7.5, 150 mm NaCl, and 0.5 mm Tris(2-carboxyethyl)phosphine. Idas-GFP, Geminin-GFP, and Geminin-HA constructs in pcDNA3.1 mammalian expression vector were described in Pefani *et al.* (24).

##### Small Angle X-ray Scattering Experiment (SAXS) Data Collection and Analysis

The three complexes purified above were used for the SAXS experiments. Three concentrations of each construct (0.5, 1.0, and 2.5 mg ml^−1^ for tGeminin-tGeminin, 1.0, 2.0, and 6.0 mg ml^−1^ for tIdas-tIdas, and 1.0, 2.0, and 6.0 mg ml^−1^ for tIdas-tGeminin) were measured at the X13 beamline at the EMBL-Hamburg, and all data were processed by PRIMUS (27) and analyzed with the ATSAS software package.

##### Analysis of the Stability of the Coiled-coil by Determination of the T*_m_*

The protein melting point (T*_m_*)^4^ of the different constructs of Idas and Geminin was determined using the Optim 1000 from Avacta. Thermal unfolding and aggregation curves were measured in 25 mm HEPES/NaOH pH 7.5, 150 mm NaCl, and 0.5 mm Tris(2-carboxyethyl)phosphine at a concentration of 1 mg ml^−1^. The T*_m_* was calculated using the ratio of fluorescence intensities at 350 and 330 nm as a function of temperature. The static light scattering signal was also recorded from the samples to detect the presence of aggregates.

##### Crystallization

The crystal screening was performed using the procedures we have previously described (Newman 2005) in 96-well sitting drop vapor diffusion plates (Greiner). After optimization, crystals used for diffraction studies were grown at 20 °C, mixing 200 nl of 10 mg ml^−1^ tIdas-Geminin with 200 nl of 0.1 m SPG buffer (pH 7.5) and 10% isopropyl alcohol. Crystals were soaked in the reservoir solution supplemented with PEG 400 to a final concentration of 30% (w/v) and vitrified by plunging into liquid nitrogen.

##### Data Collection and Structure Solution

Diffraction data were collected on the beamline ID14EH3 at the European Synchrotron Radiation Facility synchrotron (Grenoble, France) at a wavelength of 0.98 Å. The data reduction and scaling was done using the XDS package (28). The structure was solved by molecular replacement using PHASER (29) and a polyalanine model of dimeric Geminin (PDB code 2WVR) as the search model. One tIdas-tGeminin molecule was found in each asymmetric unit of the I4_1_22 cell. The model was rebuilt using COOT (30) and refined using BUSTER (31) and a local version of PDB_REDO (32) incorporating REFMAC (33). Statistics for data reduction and structure refinement are presented in Table 1.

##### Isothermal Titration Calorimetry (ITC)

The binding constants of Cdt1 (115–353) binding to tGeminin-tGeminin and tIdas-tGeminin proteins were measured by ITC (MicroCal Inc.). All samples used in the ITC experiments were dialyzed against 25 mm HEPES/NaOH pH 7.5, 150 mm NaCl, and 0.5 mm Tris(2-carboxyethyl)phosphine. The ITC measurements were performed at 25 °C by making 28 10-μl injections of the protein dimers to 1.4 ml of Cdt1. The concentrations of the Cdt1 and the Geminin complexes proteins were 0.2–10 μm and 2–80 μm, respectively. Curve fitting was performed with MicroCal Origin software.

##### Xenopus Egg Extract Assays

*Xenopus* egg extracts were prepared as described (34). Licensing assays in *Xenopus* egg extracts were performed as described in Ferenbach *et al.* (35).

##### Cell Culture, Transfection, and Immunostaining of Cells

U2OS cells were cultured in DMEM (Invitrogen) with 10% fetal bovine serum (Invitrogen). Cells were transfected with the Turbofect transfection reagent (Fermendas) according to the manufacturer's instructions.

For immunostaining, transfected cells were fixed with 4% paraformaldehyde and incubated with anti-HA (12CA5, Santa Cruz) or anti-γH2AX (Upstate) antibodies in 3% bovine serum albumin and 10% fetal bovine serum in PBS overnight at 4^ο^ C. Cells were washed with PBS 0.1% Tween and incubated for 1 h with secondary antibodies in blocking solution. DNA was stained with Hoechst.

For co-immunoprecipitation experiments (Fig. 2), U2OS cells were transfected with Geminin-GFP, Geminin-HA, and Idas-GFP or GFP and collected 24 h post-transfection. Geminin-GFP solely transfected cells served as negative control. Immunoprecipitation of Geminin-HA was performed using an anti-HA antibody (12CA5; Santa Cruz) as described in Pefani *et al.* (24). Immunoprecipitates and total cell extracts corresponding to 10% of immunoprecipitates were analyzed by Western blotting using anti-HA and anti-GFP (Molecular Probes) antibodies.

## RESULTS

### 

#### 

##### The Idas-Geminin Heterodimer Is as Stable as Geminin-Geminin Homodimers, in Contrast to the Unstable Idas-Idas Homodimer

**FIGURE 1.**
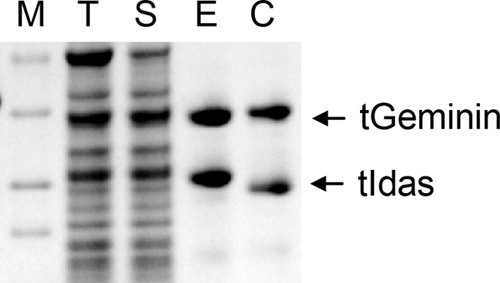
**Idas and Geminin form a stoichiometric complex after co-expression of Geminin and Idas in bacteria and purification by using immobilized metal affinity chromatography (IMAC).** The SDS gel shows the protein marker (*M*), total cell extract (*T*), soluble fraction (*S*), elution (*E*), and elution fraction after cleavage of the his tag (*C*).

The coiled-coil domains of Geminin and Idas can assemble in homodimers, and Geminin and Idas can also interact with each other through their coiled-coil domain (24). We have also shown that Idas prefers to interact with Geminin (24). To study the three possible complexes between Geminin and Idas (Geminin-Geminin, Idas-Idas, and Idas-Geminin), we expressed and purified both the coiled coil regions (tGeminin comprising residues 82–160 and tIdas comprising residues 173–245) or longer versions (dGeminin residues 29–201, lacking the N-terminal 28 residues, and dIdas, comprising residues 101–284, as longer constructs of Idas proved to be insoluble). Idas and Geminin, both, formed homodimers when expressed on their own, as expected. An Idas-Geminin complex was formed by co-expression in *Escherichia coli* and was purified using a hexahistidine tag on Idas (Fig. 1).

**FIGURE 2.**
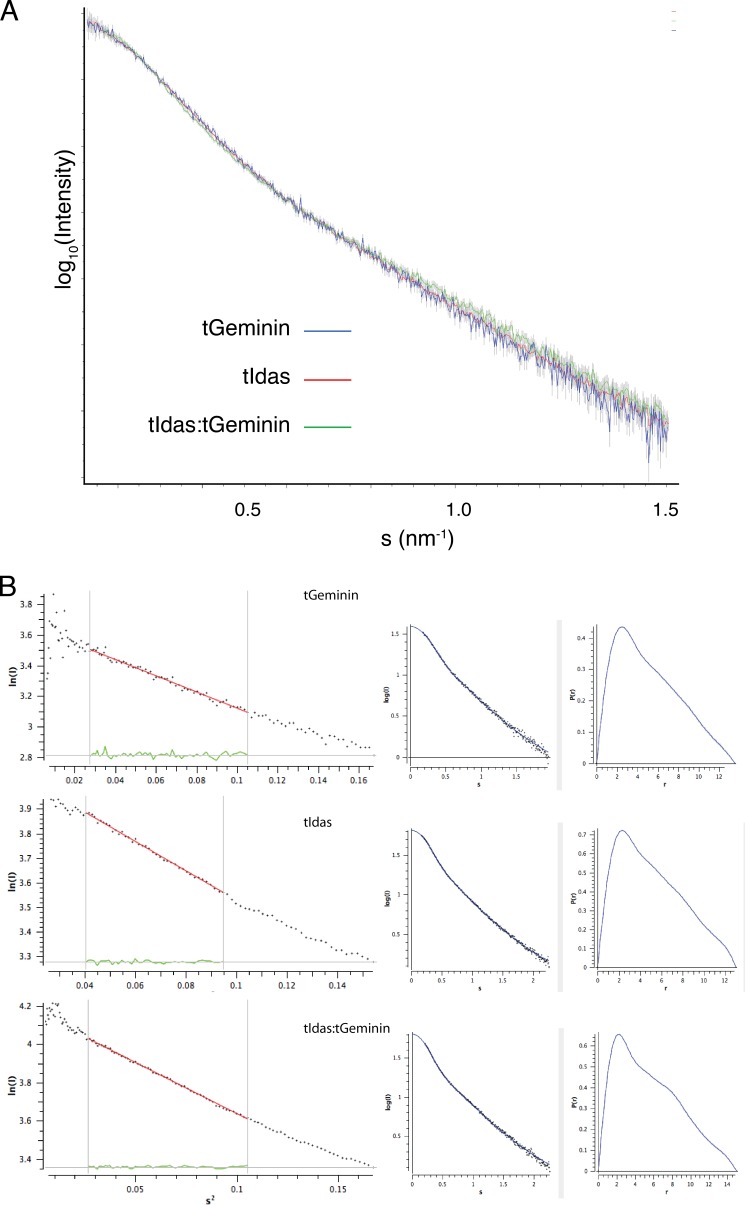
**SAXS analysis of Geminin, Idas, and the Idas-Geminin complex.** *A*, the scattering curves for tGeminin, tIdas, and tIdas-tGeminin complexes; only the low angles are shown for clarity. *B*, Guinier plots and distance probability distribution functions for the same complexes.

To further characterize the three complexes in solution, we performed a SAXS. All three complexes were tested in three different concentrations, and the x-ray scattering data were subsequently analyzed with the ATSAS package (36). The scattering curves for all three complexes are very similar to each other (Fig. 2*A*), and both the Guinier plot analysis and the distance probability distribution function (Fig. 2*B*) showed that all three complexes had similar radii of gyration (4.0–4.2 nm) and similar maximal distances (14–15 nm), arguing that all three are dimers and have a similar shape in solution.

**FIGURE 3.**
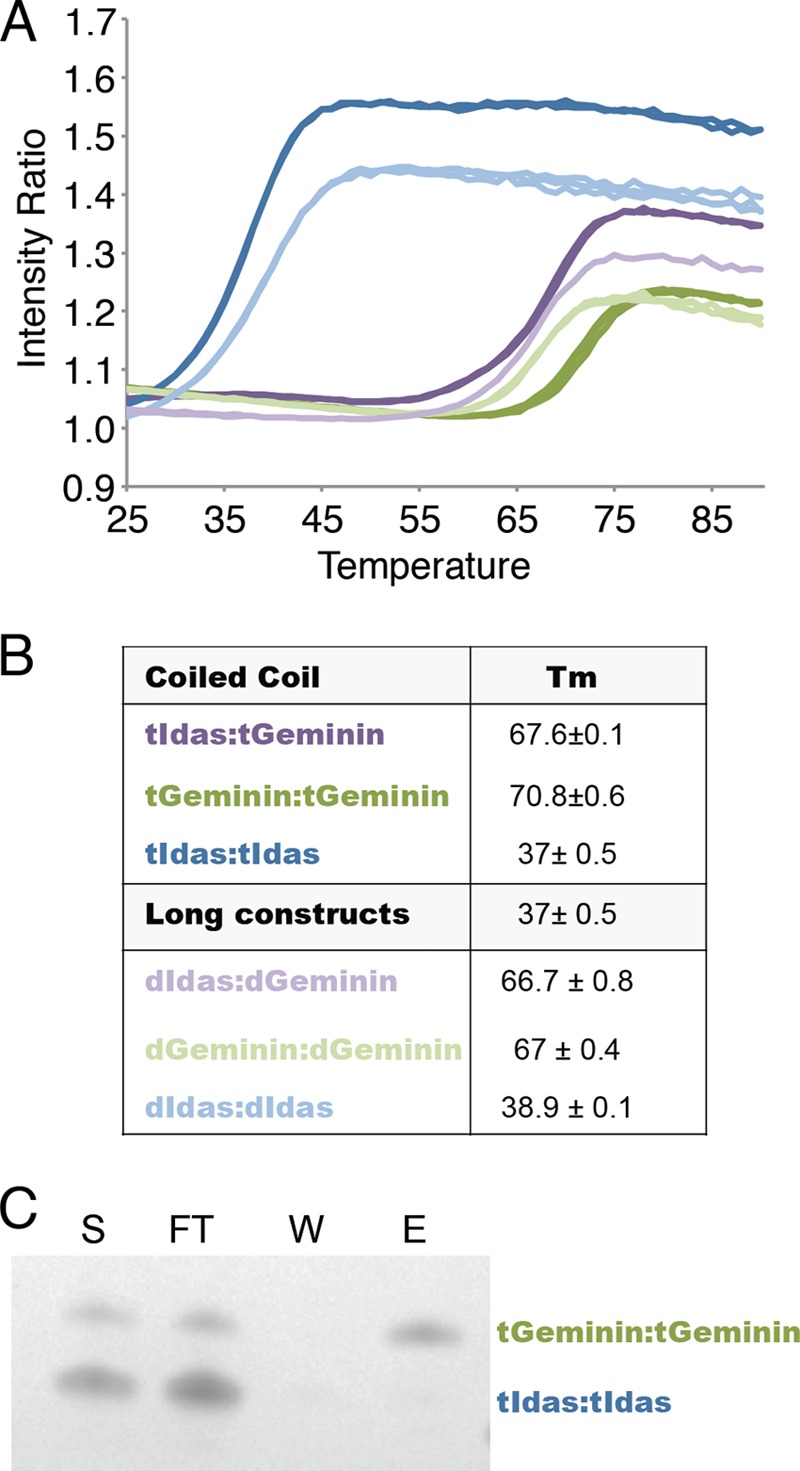
**Stability analysis of the different Idas and Geminin dimers.** *A*, the ratio of fluorescence intensities at 350 and 330 nm (indicative of folding) as a function of temperature, used to calculate the T*_m_* valueσ. *B*, the T*_m_* values derived from the experimental data. *C*, a pulldown assay on the purified homodimers shows that the association of the tGeminin dimers is irreversible. Purified his-Geminin:tGemnin was mixed with purified tIdas-tIdas (*S*) and immobilized on Talon beads. The different fraction are: flow-through (*FT*), wash (*W*), elution (*E*).

Next, we analyzed the stability of these dimeric complexes by thermal denaturation using the OPTIM 1000 instrument (Fig. 3, *A* and *B*). First, we analyzed the stability of dimerization of the coiled coil regions. The tGeminin homodimer had a T*_m_* of 70.8 °C, significantly higher than the value of 35 °C previously reported for a smaller peptide comprising residues Leu-110–Ala-145 of Geminin (37). This suggests that the first and second heptads (Geminin 96–109) are very important for the stability of the coiled-coil. The tIdas homodimer (T*_m_* = 37 °C), in contrast, is very unstable. Interestingly though, the stability of the tIdas-tGeminin complex (T*_m_* = 67.6 °C) is almost identical to that of the tGeminin homodimer (T*_m_* = 70.8 °C). Similar values were obtained when testing for stability longer constructs of each dimer using dGeminin and dIdas.

These agree well with our previously shown data that Idas prefers to associate with Geminin when both are available as GFP labeled proteins in transfected cells (24), implying that the Idas homodimer is less stable. Moreover, the actual T*_m_* of 37 °C for the Idas homodimer also indicates that it is probably very unstable *in vivo*, and thereby Idas alone would probably only exist in a dynamic equilibrium between the monomeric and dimeric form in cells. In the presence of Geminin, Idas should readily associate with it to form the far more stable Idas-Geminin heterodimer.

In contrast, both the Geminin homodimer or Idas-Geminin dimers are so stable that their dissociation rate would be expected to be very low. Indeed, pulldown assays show that if the purified tGeminin and tIdas homodimer are mixed together they do not associate into a tIdas-tGeminin heterodimer (Fig. 3*C*). Thus, the choice of the partner is likely to take place close to translation, whereas both proteins exist as monomers.

##### Geminin Prefers to Heterodimerize with Idas in Human Cells

**FIGURE 4.**
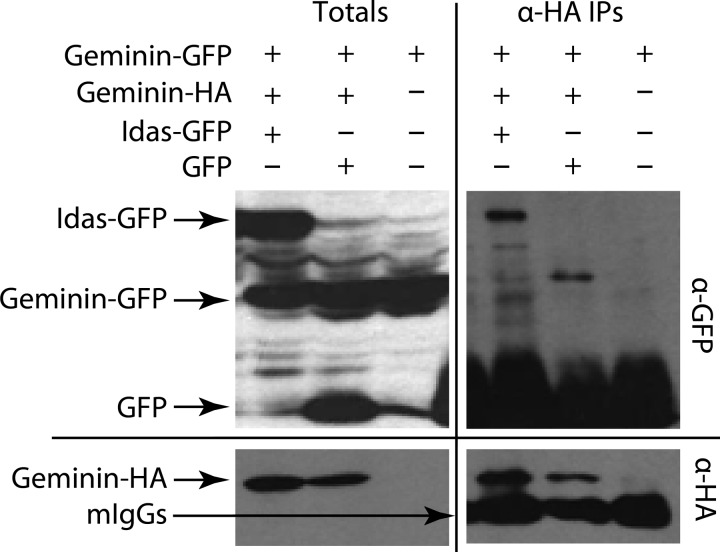
**Idas is the preferred dimerization partner for Geminin.** U2OS cells were (co)transfected with full-length Geminin-GFP, Geminin-HA, and/Idas-GFP or GFP as indicated. Cells transfected with GemininGFP alone served as the negative control. Total cell lysates (*left*) and anti-HA immunoprecipitates (*IP*, *right*) were immunoblotted for GFP (*upper*) and HA (*lower*). When both Idas and Geminin were available in similar amounts, Geminin preferred to heterodimerize with Idas.

The stability data above as well as data we have previously shown in co-transfection and immunoprecipitation experiments (24) clearly indicate that Idas interacts preferentially with Geminin rather than with itself. But does Geminin also interact preferentially with Idas? The previous experiments showed that this choice is most likely co-translational, as preformed dimers do not re-associate to new ones. To assess preferential complex formation in cells, we transfected U2OS cells with constructs expressing full-length Geminin-HA, Geminin-GFP, and Idas-GFP or GFP as a control (Fig. 4). Although transfected Geminin-HA is able to co-precipitate Geminin-GFP, in the presence of Idas-GFP, Geminin-HA co-precipitates Idas-GFP, and no Geminin-GFP is detectable in the immunoprecipitate. This finding strongly indicates that in U2OS cells, when both proteins are expressed to comparable levels, Geminin prefers to form a dimer with Idas than with itself. We conclude that both Geminin and Idas prefer to form heterodimers with each other than homodimers with themselves when expressed to comparable levels.

##### The Crystal Structure of the Idas-Geminin Coiled Coil Dimmer

The above data collectively suggest that an Idas-Idas homodimer is unlikely to exist in cells and that Idas is very likely to sequester Geminin when both proteins are available in the cell. Thus the Idas-Geminin heterodimer is likely to be the functional form of Idas. To understand the Idas-Geminin interaction in further detail, we crystallized the tIdas-tGeminin complex we described above and determined its structure by x-ray crystallography. The structure was refined against 2.9 Å data to an *R*_free_ of 22.6% and is of excellent quality, contains no Ramachandran outliers, and ranks in the best 99th percentile compared with structures determined at similar resolution in Molprobity (38). Crystallographic statistics are shown in Table 1.

**TABLE 1:** **Crystallographic data collection and refinement statistics**

**Data collection**	
Space group	I 41 2 2
Cell dimensions, *a*, *b*, *c* (Å)	117.80, 117.80, 103.46
Resolution (Å)	46.95-2.89 (3.05-2.89)
*R*_merge_	0.128 (0.839)
*I*/σ*I*	9.8 (2.3)
Completeness (%)	99.8 (99.5)
Redundancy	6.3 (6.4)

**Refinement**	
Resolution (Å)	83.30-2.89
No. reflections	7765
*R*_work_/*R*_free_	20.3/22.6
No. atoms	
Protein	1139
Ligand	36
*B*-Factors (Å^2^)	
Wilson	81.3
Average of atoms	53.1
r.m.s.d.	
Bonds (Å) r.m.s.d./r.m.s.Z	0.018/0.945
Angles (°) r.m.s.d./r.m.s.Z	2.296/1.048

**Validation (Molprobity)**	
Ramachandran favored	100%
Ramachandran outliers	0%
MolProbity score	1.95

**FIGURE 5.**
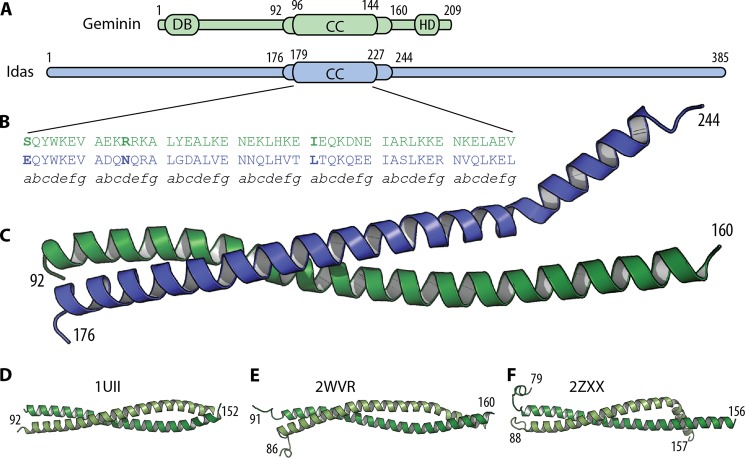
**The structure of the Idas-Geminin complex.** *A*, a diagram of the domain structure for Geminin and Idas·coiled-coil domain (*CC*), destruction box (*DB*), homeodomain binding region (*HD*). The limits of the crystallization construct (tIdas-tGeminin) and those of the coiled coil are *numbered* and denoted as *thicker boxes. B*, a sequence alignment of the seven heptads involved in the coiled coil. *C*, a schematic representation of the crystal structure of the tGeminin-tIdas structure. Differences in the *a* and *d* register positions are in *bold. D–F*, the structures of the Geminin homodimer, alone (*D*) and from the complex with Cdt1 (*E* and *F*).

The Idas and Geminin coiled-coil regions form a head to head (parallel) heterodimer (Fig. 5). The secondary structure is α-helical from residues Pro-94 to Asn-159 for Geminin and from Pro-178 to Leu-241 for Idas. The overall structure is similar to the coiled-coil domain of the Geminin-Geminin homodimer (Fig. 5, *D–F*). The root mean square deviation (r.m.s.d.) of the Idas to the Geminin monomers is 4.2 Å when the two monomers are compared as single rigid bodies but has an r.m.s.d._f_ of 1.4 Å when compared as two rigid bodies each, as optimized by the RAPIDO algorithm (39). Idas has an r.m.s.d._f_ of 0.8 and of 1.2 Å when compared with each of the two Geminin monomers of the Geminin homodimer structure (19) (PDB code 1UII). Geminin from the Idas-Geminin complex had an r.m.s.d._f_ of 1.5 and 1.1 Å compared with the same two Geminin monomers of the homodimer. This suggests that when our heterodimer structure is compared to the Geminin homodimer structure, Geminin in the heterodimer resembles the second Geminin monomer of the homodimer (PDB code 1UII, chain B), and Idas resembles the first Geminin monomer (PDB code 1UII, chain A). That information has been used in all subsequent superpositions presented here, where Geminin is always superposed to the second monomer of the 1UII homodimer. This is also reflected in our preference to refer to an Idas-Geminin heterodimer in the context of this study.

The coiled-coil domain includes residues 96–144 of Geminin and 179–227 of Idas. The α-helix of Idas is kinked at the position Ala-228, a position comparable to the kink in one of the Geminin-Geminin homodimer structures (*2ZXX*, Fig. 5*F*). The bent part of the coiled coil in the Idas-Geminin structure is involved in crystallographic contacts, as in the 2ZXX structure, and is most likely induced by crystallographic packing. Although this C-terminal region is helical, it does not follow the typical coil structure.

**FIGURE 6.**
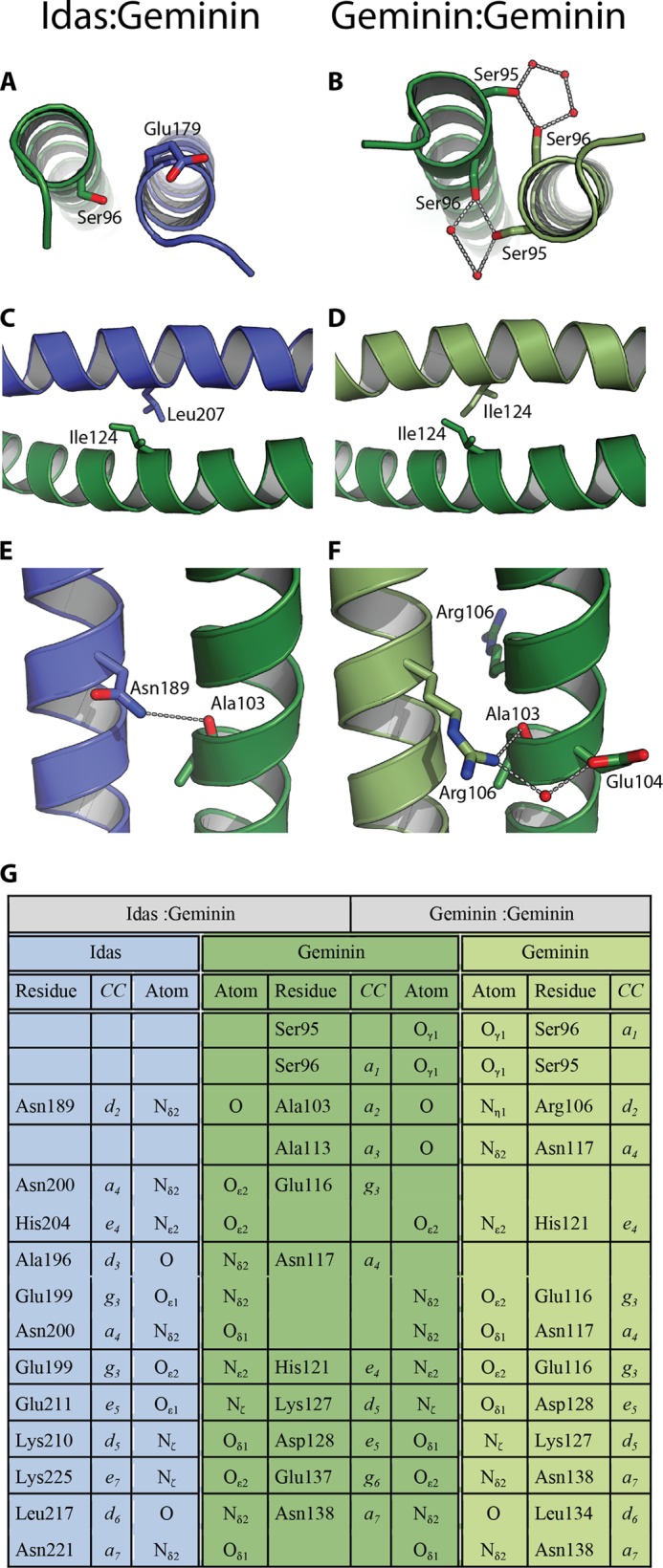
**Interactions in the coiled coil dimerization interface.** Shown are the interactions in the *a_1_*, *a_5_*, and *d2* positions in the tIdas-tGeminin complex (*A*, *C*, and *E*) and in the tGeminin-tGeminin complex (*B*, *D*, and *F*). *G*, a lookup table for all non-hydrophobic interactions of coiled coil region for the tIdas-tGeminin complex (*left half*) and the tGeminin-tGeminin complex (*right half*). The residue names and numbers, coiled coil register positions, and atom names are indicated.

The coiled-coil domain is composed of seven heptads denoted as (*abcdefg*)_1–7_ (Fig. 5*B*). Along these seven heptads, Geminin (96–144) and Idas (179–227) share 51% sequence identity. Coiled coils are typically viewed to have two register positions, *a* and *d*, comprising a hydrophobic core interface. Neither Geminin nor Idas are typical coiled coils in that respect. Only four and five of the seven residues in the a and d positions, respectively, are hydrophobic in Geminin and the same stands for Idas. Of the 14 total residues in these two positions, only three are different between Idas and Geminin (Fig. 6).

The first difference is in the *a* position of the first heptad (*a*_1_) that is a hydrophilic residue both in Geminin and Idas. Although Geminin has in the *a*_1_ position Ser-96, Idas has in the equivalent position Glu-179 (Fig. 6, *A* and *B*). In the Geminin homodimer (PDB code 1UII), the γ-hydroxyl of Ser-96 of each monomer is implicated in an intricate hydrogen bonding network with two neighboring water molecules and the preceding Ser-95 of the other molecule. That network should contribute to stabilize the N-terminal region of the coiled-coil. In the Idas-Geminin complex, Glu-179 of Idas points toward the solvent and is unlikely to affect the heterodimer stability. In a putative Idas-Idas homodimer, the two glutamates could create electrostatic repulsion. Interestingly, a thermal stability study on a-a′ pairs of short coiled-coils has shown that a Glu-Glu a-a′ pair has a destabilizing coupling energy and encourages heterodimerization (40).

The second difference is located in the fifth heptad and is the hydrophobic *a*_5_-*a*_5_′ pair Ile-124–Ile-124 in the Geminin homodimer (Fig. 6, *C* and *D*). This position is Leu-207 in Idas; in the Idas-Geminin complex the pair becomes Leu-207–Ile-124 and is packed well in the structure (Fig. 6*D*). The Leu-207–Leu-207 pair in the Idas-Idas dimer would be expected to be equally stable; indeed a computational mutagenesis experiment using either the Geminin homodimer or our Idas-Geminin structure and mutating this specific position to have the Leu-Leu pair using FoldX results in very small differences in the change of the free energy of folding (ΔΔ*G* of 0.18 and −0.41 kcal mol^−1^, respectively).

The third difference is in the *d*_2_ position and involves again non-hydrophobic residues, Arg-106 in Geminin that becomes Asn-189 is Idas (Fig. 6, *E–F*). In the Geminin homodimer structure, Arg-106 of the first monomer makes two important hydrogen bonds with the second monomer; one with the main chain carbonyl oxygen of Ala-103 and one with a water molecule that bridges to the carboxyl group of Glu-104. Asn-189 of Idas bridges with the main chain carbonyl oxygen of Ala-103 of Geminin, similar to what was observed for the Geminin Arg-106 side chain (an observation that reinforces our previous observations that Idas resembles more the first monomer of the Geminin homodimer structure).

Τhe very characteristic network of hydrophilic interactions between the Geminin homodimer (PDB code 1UII) and the Idas-Geminin heterodimer is largely similar (Fig. 6*G*), although the interacting residues are sometimes slightly different (*e.g.* glutamate instead of an aspartate). Besides the loss of the Ser-95–Ser-96 interaction we already discussed, the only other differences are in the neighborhood of Glu-116–Asn-117 in Geminin, which is largely rearranged but results in a net “win” of a single hydrogen bond in the Idas-Geminin heterodimer.

In conclusion, the differences between the structure of the Geminin homodimer and the Geminin-Idas heterodimer lead to equally stable dimers, in agreement with the experimental data. As the structure of the Idas-Geminin heterodimer is stable and some residues of Geminin involved in interactions with Cdt1 would still be available, we decided to proceed with characterizing its interaction with Cdt1*in vitro*.

##### The Interaction of Idas with Geminin Affects Cdt1 Binding in Vitro

**FIGURE 7.**
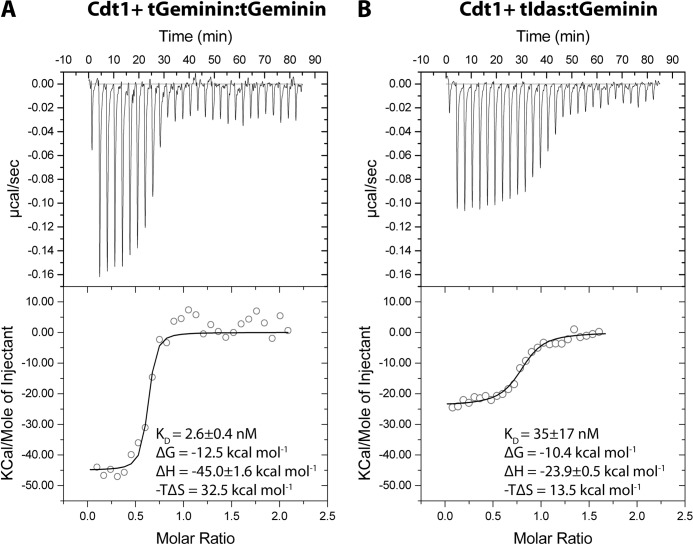
**The tIdas-tGeminin complex binds Cdt1 with less affinity and different thermodynamic characteristics than the tGeminin-tGeminin.** *A* and *B*, isothermal titration calorimetry data recorded upon successive injections of tGeminin-tGeminin or tGeminin-tIdas into a cell containing tCdt1 and the analysis. The derived values for the *K_D_* and the change in free energy (Δ*G*), enthalpy (Δ*H*), and entropy (−ΤΔ*S*) are shown; the small difference in the change of the free energy of binding in fact results from large changes in the relative contribution of enthalpy and entropy.

We previously reported that Idas alone does not bind Cdt1 (24), whereas the affinity of an Idas-Geminin complex for Cdt1 is significantly reduced compared with Geminin based on surface plasmon resonance experiments. Here we used ITC to analyze the binding of the three dimers, tGeminin-tGeminin, tIdas-tGeminin, and tIdas-tIdas, to a truncated Cdt1 construct spanning residues 113–353 in solution and in more detail (Fig. 7). We measured that the affinity of tIdas-tGeminin for Cdt1 is about 35 nm, ∼10-fold lower than the affinity of the Geminin homodimer for Cdt1 (*K_D_* ∼ 3.5 nm).

To confirm that Cdt1 binds to the heterodimer and not to a fraction of Geminin homodimer, equimolar amounts of purified tCdt1 and purified tGeminin-tIdas were mixed and tested by size exclusion chromatography for complex formation. The elution profile shows that tCdt1 binds to the heterodimer tGeminin-tIdas. This experiment further strengthens our previous observations that once assembled, the coiled coil dimers are unlikely to disassemble and form homodimeric complexes (data now shown).

It should be noted that the affinity of the tIdas-tGeminin heterodimer for tCdt1 is still high, suggesting that Idas-Geminin could interact with Cdt1 *in vivo*. The thermodynamic analysis of complex formation is also of interest. The modest difference in the free energy of binding (2.1 kcal mol^−1^) comes from substantial changes in the entropy and the enthalpy of the system. The enthalpy for binding is in both cases favorable for complex formation but is much less favorable (by 21.1 kcal mol^−1^) for the tIdas-tGeminin binding. This is easily explainable, as Idas lacks many of the residues that interact with Cdt1, and specific interactions are lost in the tIdas-tGeminin complex. The entropy in both cases is unfavorable for complex formation (likely due to a loss in Cdt1 conformational freedom, resulting in a negative change in conformational entropy) but much less unfavorable (by 19.5 kcal mol^−1^) for tIdas-tGeminin binding. The lesser entropy change upon tIdas-tGeminin binding could be due to the formation of a smaller interface between Geminin-Idas and Cdt1 and, thus, lesser conformational changes. Thus, these two opposing effects compensate each other, resulting in an apparent difference in affinity of about 10-fold.

##### The Interaction of Idas with Geminin Affects Geminin Function in Xenopus Egg Extracts

**FIGURE 8.**
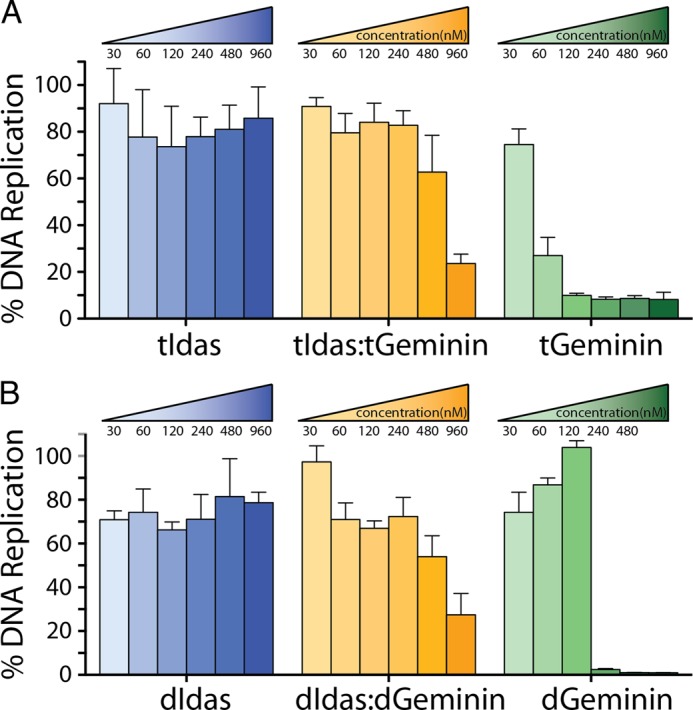
**Idas inhibits Geminin function as an inhibitor of DNA replication in *Xenopus* egg extracts.** *A*, increasing concentrations of tIdas-tIdas, tIdas-tGeminin, and tGeminin-tGeminin were assessed for their ability to inhibit DNA replication. DNA replication is presented as a percentage relative to 100% replication of buffer treated extract. S.E. are derived from three independent experiments. *B*, the same for the dIdas and dGeminin constructs.

Having established that Idas-Geminin interacts with Cdt1, albeit with lower affinity compared with Geminin-Geminin, we next wanted to study the biochemical function of the different complexes, Geminin-Geminin, Idas-Geminin, and Idas-Idas, in their ability to inhibit replication licensing and subsequent replication of sperm DNA in a *Xenopus* egg extract (Fig. 8). The assay in *Xenopus* egg extract allows measuring the activity of the purified homo or heterodimers in a system that supports DNA replication under the same control mechanisms as in early embryonic cells (41). First we used the coiled-coil domain alone (Fig. 8*A*), tGeminin and tIdas, as it has been shown that this truncated Geminin construct is competent for efficiently inhibiting licensing (21). tGeminin showed efficient inhibitory activity on DNA replication, with an IC_50_ of ∼50 nm, consistent with previous studies (20). The tIdas-tGeminin heterodimer inhibited DNA replication less efficiently, with an IC_50_ of ∼720 nm. Finally, the tIdas homodimer had no effect on DNA replication. We confirmed these results using the longer constructs of Geminin and Idas, dGeminin and dIdas. These constructs have similar inhibitory activities as the shorter forms (Fig. 8*B*). These findings correlate well with the lower affinity of tIdas-tGeminin for Cdt1 that we observed *in vitro*.

From these experiments we conclude that the stable and preferred heterodimer, which should be assembled post-translationally by Idas and Geminin monomers binding together, is still able to inhibit DNA replication but with an efficiency about 15 times less than that of the Geminin homodimer.

##### Idas Affects Geminin Function in Mammalian Cells

**FIGURE 9.**
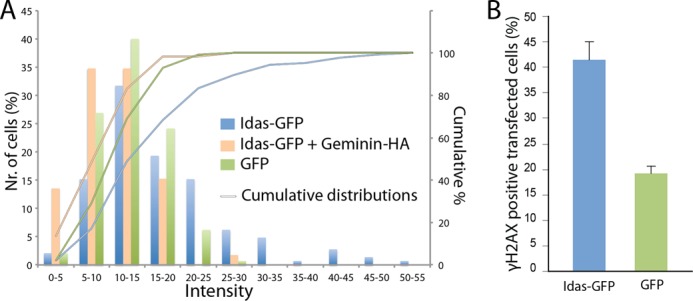
**Overexpression of Idas in U2OS cells results in increased DNA content.** *A*, full-length Idas-GFP, Idas-GFP and Geminin-HA, or GFP were transfected in U2OS cells. 52 h post transfection cells were fixed, and DNA was stained with Hoechst. DNA content in each transfected cell was quantified by measuring the total intensity of Hoechst staining per nucleus using Image J. The results are presented both as a histogram (bars showing the percentage of cells within a range of arbitrary intensity units) and as a cumulative distribution (lines showing the percentage of cells below a certain intensity). Idas-GFP positive cells show an increased number of cells with high Hoechst intensity compared with the GFP control; co-expression of Geminin decreases the number of cells with high DNA content both compared with Idas and to the GFP control. *B*, U2OS cells transfected with Idas-GFP and GFP were stained for γH2AX, which marks double-strand breaks. Idas-GFP transfected cells present an increased percentage of cells staining positive for γH2AX compared with cells transfected by GFP alone, indicating over-replication. *Error bars* are derived from three independent experiments.

To confirm the ability of Idas to affect Geminin function in DNA replication in mammalian cells, we used U2OS cells, where Geminin depletion has been shown to cause DNA over-replication (42–44). To this end, we overexpressed full-length Idas-GFP in U2OS cells, and 52 h post transfection cells were fixed and DNA was stained with Hoechst. After transient transfections, Idas mRNA was increased by several hundred-fold (data not shown). The total Hoechst intensity per nucleus was quantified using the ImageJ software. The number of cells with a high level of intensity/nucleus was found to be significantly higher in Idas-GFP compared with GFP transfected cells, indicative of increased DNA content in these cells (Fig. 9*A*). Idas-GFP-transfected nuclei were also stained positive for γH2AX, a double-strand break marker that has been connected with DNA over-replication events (Fig. 9*B*).

The over-expressed Idas is likely to sequester endogenous Geminin as it is produced to form the Idas-Geminin preferred heterodimer. Excess Idas would be expected to partially form Idas homodimers, which would be unstable and unable to affect replication. Under these conditions we expect most of endogenous Geminin to be in a complex with Idas. As this Idas-Geminin complex can still inhibit replication licensing, albeit about 15 times less efficiently as indicated in the *Xenopus* experiments, we would expect a mild phenotype compared with Geminin depletion, as we observes (Geminin depletion results in a much larger increase in DNA content in these cells as expected; data not shown). To confirm this model, we then decided to check the effect of co-expressing Geminin together with Idas. If the effect of Idas over-expression was mediated through binding to endogenous Geminin, we would expect the co-expression of exogenous Geminin to alleviate the over-replication phenotype. Indeed, when Geminin-HA was co-expressed together with Idas-GFP, per nucleus DNA content was shifted toward the normal GFP control levels (Fig. 9*A*). The slight shift toward less DNA content than the GFP control experiment is likely due to the formation of some Geminin homodimers due to excess Geminin in some cells, which would strongly suppress replication below control levels as has been previously shown in the same cell line as used for our experiments (45). These observations also support a model where the sequestration of Geminin into the Idas-Geminin heterodimer lowers the inhibitory activity of Geminin on licensing.

## DISCUSSION

We present the first structure of a heterodimer between the coiled coil region of Geminin and Idas (also called Multicilin). Idas and Geminin coiled-coil regions form a head-to-head heterodimer in solution. The crystal structure analysis suggests that Idas and Geminin form a very tight heterodimeric complex with similar stability to that of the Geminin-Geminin homodimer. Before this work, Geminin had been shown to exist, bind to other proteins, and function as a tight homodimer. Here we show that an Idas-Geminin heterodimer is very stable and appears to be the preferred state when both proteins are co-expressed in cells. Despite the many binding partners reported for Geminin, Idas is to our knowledge the first protein shown to be able to replace a second molecule of Geminin in forming a coiled-coil. Given the many binding interfaces offered by Geminin outside the coiled-coil domain (and the importance of these interactions for Geminin function in both cell cycle and differentiation), the structure presented here offers novel insight into the regulation of Geminin and the function of Idas in cells.

Biophysical stability analysis showed that the Geminin homodimer and the Idas-Geminin complexes have similar thermal stability, unlike the Idas homodimer, which has a T*_m_* of 37 °C and is most likely in a dynamic monomer to dimer equilibrium in cells. We previously showed that Idas prefers to interact with Geminin than itself (Pefani *et al.* (24)), and here we show that Geminin also prefers to interact with Idas than itself when both proteins are co-expressed in cells. This preference may be affected by relative levels of expression (during the cell cycle and in different cell types) and by post-translational modifications of the coiled-coil domains but clearly suggest that Idas interaction with Geminin is important for function. The many functions of Geminin, as we discuss below in further detail, could be modulated by dimerization with Idas. In addition, given the low relative abundance of Idas in most cell types (Pefani *et al.* (24)) and the highly unstable nature of the Idas homodimer, we would expect that all of Idas would be in complex with Geminin in most cells, and its function would be performed through an Idas-Geminin complex.

We also show that the Geminin homodimer is very stable and probably has a very slow rate of dissociation. Once it is assembled, Idas is not able to form heterodimers with Geminin, at least *in vitro*. Only when both proteins are co-expressed do they assemble into a heterodimer, suggesting that Idas can only interact with newly synthesized, but not pre-existing, Geminin. Although Geminin is a stable protein *in vitro*, it is known to be unstable *in vivo* due to proteolysis. Geminin starts accumulating at the G_1_/S phase transition and continues during S phase (46). Geminin levels persist through the S and G_2_ phases, and the protein is degraded in anaphase through anaphase promoting complex/cyclosome-mediated proteolysis (7). Furthermore, in S phase the level of Geminin is constant but is constantly degraded and synthesized with a turnover of 3–4 h (47). Thus newly synthesized Geminin is almost constantly present to interact with Idas.

We could show that Idas is able to suppress Geminin-mediated inhibition of DNA replication both in *Xenopus* egg extract and in mammalian cells. The Idas homodimer has no effect on DNA replication, but the Idas-Geminin complex is less able to inhibit DNA replication licensing, in effect inhibiting Geminin activity. Affinity measurements of Geminin homodimer and Idas-Geminin heterodimer binding to Cdt1 in solution show that the heterodimer has a 10-fold lower affinity for Cdt1, which correlates well with the results observed in *Xenopus*. Together these results suggest that the sequestration of Geminin into the Idas-Geminin heterodimer is responsible for suppression of the Geminin inhibitory function when Idas is overexpressed.

The lower affinity of the Idas-Geminin heterodimer for Cdt1 compared with the Geminin homodimer also means that Idas must sequester all or most of Geminin to affect its function. High levels of Idas have been previously reported in the choroid plexus and the cortical hem of the mouse telencephalon (24). In this specific part of the brain Geminin is predominantly expressed by neural progenitors (48), whereas Idas levels become higher in specific differentiated cell types (Pefani *et al.* (24)). High levels of Idas have also been reported in other developing multiciliated cells (Stubbs *et al.* (25)). Recent studies suggest that Geminin participates in the acquisition of neural cell fate by embryonic stem cells (49) and regulates self-renewal and differentiating decisions of cortical neural progenitor cells (50). Moreover, it has been previously suggested that balanced interactions between Geminin and Cdt1 or Geminin and Six3 or Homeobox-containing transcription factors might affect cellular decision toward proliferation and differentiation (17, 18). Therefore, Idas expression could modulate Geminin activity in progenitor cells promoting specific differentiation pathways.

The function of the interaction of Idas with Geminin could also be to recruit Idas to Cdt1. Several functions have been attributed to Cdt1 such as loading of the MCM complex to the pre-replication complex (51, 52), chromatin unfolding via opposing functions of HBO1 and HDAC11-geminin (53, 54), DNA repair (55), and more recently in mitosis as Cdt1 is required for the correct attachment of the microtubules to the kinetochore (56). Idas was recently identified as a key regulator of multiciliate cell differentiation by coordinately promoting cell-cycle exit, deuterosome-mediated centriole assembly, and FoxJ1 expression (25). It is likely that the multiple functions of Geminin and Idas are coordinated and/or regulated by the Idas-Geminin complex, which appears to be the preferred state of both proteins when both are available in a cell. Its important to note that many origin licensing proteins have recently been implicated in cilia formation and the etiology of Neier-Gorlin syndrome (57). This might suggest that the established role of Idas in multiciliate cell differentiation could also be through modulation of the licensing activity of Geminin and not only through transcriptional modulation. Our observations on the importance of the Idas-Geminin heterodimer also raise the possibilities that other Geminin homologues, like the replication factor GEMC1, form similar complexes, mediating their recruitment or regulating functions of Geminin.

## Footnotes

4 The abbreviations used are:

T*_m_*: melting point
ITC: isothermal titration calorimetry
SAXS: small angle x-ray scattering experiment
r.m.s.d.: root mean square deviation.
